# Effect of robot for medication management on home care professionals’ use of working time in older people’s home care: a non-randomized controlled clinical trial

**DOI:** 10.1186/s12913-023-10367-0

**Published:** 2023-12-02

**Authors:** Satu Kajander-Unkuri, Mojtaba Vaismoradi, Jouko Katajisto, Mari Kangasniemi, Riitta Turjamaa

**Affiliations:** 1 Department of Nursing Science, Faculty of Medicine, University of Turku, Turku, Finland; 2 Diaconia University of Applied Sciences, Helsinki, Finland; 3 Faculty of Nursing and Health Sciences, Nord University, Bodø, Norway; 4 Department of Mathematics and Statistics, University of Turku, Turku, Finland; 5 Satasairaala, Pori, Finland; 6 Savonia University of Applied Sciences, Kuopio, Finland; 7 Faculty of Science and Health, Charles Sturt University, Orange, NSW Australia

**Keywords:** Home-care professional, Medication management, Older people’s home care, Patient safety, Robot, Working time

## Abstract

**Background:**

Medication management has a key role in the daily tasks of home care professionals delivered to older clients in home care. The aim of this study was to examine the effect of using a robot for medication management on home care professionals´ use of working time.

**Methods:**

A pragmatic non-randomized controlled clinical trial was conducted. The participants were home care professionals who carried out home care clients’ medication management. Home care clients were allocated into intervention groups (IG) and control groups (CG) (n = 64 and 46, respectively) based on whether or not they received the robot. Data were collected using the Working Time Tracking Form prior to and 1 and 2 months after introducing the intervention. The t-test was used to compare the groups at each three timepoints. Analysis of Covariance was used to examine the groups’ differences for the total time for medications as the number of visits per day as the covariate.

**Results:**

With robot use, the total amount of home visits decreased by 89.4% and 92.4% after 1 and 2 months of intervention use, respectively, compared to pre-intervention (p < 0.001). The total working time used for medication management considering the number of visits per day decreased from 54.2 min (95% CI 49.6–58.8) to 34.9 min (31.4–38.3), i.e., by slightly over 19 min (p < 0.001) in the IG group. During the follow-up, the total working time used for medication management considering the number of visits per day remained the same in the CG group.

**Conclusion:**

Using a robot for medication management had a notable effect on decreasing the use of working time of home care professionals. For health services, decreased use of working time for medication management means that the time saved can be assigned to services that cannot be replaced otherwise. More digital solutions should be developed based on home care clients’ and professionals’ needs to meet the challenge of the growing number of older people in need of home care and ensure their safety.

**Trial registration:**

ClinicalTrials.gov Identifier: NCT05908604 retrospectively registered (18/06/2023).

## Background

In older clients’ home care, medication management has a key role in the daily tasks of home care professionals [1, 2]. In addition, the professionals take care of older home care clients with personal assistance for eating and nursing treatments such as wound care [2]. Typically, older people use between one and five separate medications every day. Medication administration and ensuring that medicines are taken at the right time is an important and time-consuming task in daily medication management for home care professionals. It is noteworthy that for many older clients, securing medication is the only reason for the home visits [1, 2]. At the same time, most of them also use self-care medication that can be bought without a prescription [3, 4]. Therefore, they are involved in managing their own medication regimens in their everyday lives [4, 5].

In home care, medication management is a process referring to the ordering, dispensing, reconstitution, administration, and monitoring of the effects of medications and medication education [6, 7]. Home care professionals take care of medication management by ordering medications from a pharmacy. After that, the professionals dispense medications manually to the clients’ dosette for one week. It is important for home care clients to take medication at the right time to achieve the best possible benefit and, on the other hand, to avoid inappropriate side effects if the medicines are taken at the wrong time. Home care clients can sometimes take their medications by themselves or with the help of their families and relatives [8, 9].

During daily home visits, home care professionals are obliged to monitor the effects of medications by following up the health condition of older home care clients using different measurement methods such as the measurement of blood pressure. In addition, home care clients should be asked about the effects and adverse effects of medication [10]. Safety of medication is a central element in home care. During home visits, home care professionals prevent polypharmacy, the over-prescription of medications, and medication errors to ensure home care clients’ safe living at home [11]. Medication education is an important task of home care professionals [12]. Home care professionals improve clients’ understanding and adherence to medications by educating them about the indications and common and severe adverse effects of medication [13, 14], especially those receiving high-risk medications [14]. Different digital solutions have been developed to ensure medication safety while reducing the workload of home care professionals [8, 15, 16]. These include robots for medication management that remind home care clients to take their medicines at the proper time [16], thus relieving professionals of this task [8].

Nurses’ use of working time in hospitals has been studied from the viewpoint of the use of the robots delivering nursing care, such as auto-tracking systems to identify patients and robots taking care of patients’ hygiene. These studies found that the use of robots for different nursing treatments decreased the nurses’ use of working time required per patient when compared to manual care realized by the nurses [17].

There is a lack of studies related to home care professionals’ use of working time in older people’s home care, and especially in medication management. The use of robots for medication management by older people’s home care has been studied in the past, but these studies have mostly focused on testing different robots from the technical point of view [18, 19]. The number of older people receiving home care is increasing [20] as are the number of clients receiving multiple medications that require assistance to administer. At the same time, the shortage of home care professionals is growing [21]. Therefore, it is necessary to investigate potential new possibilities, such as robots, to decrease the workload of homecare professionals and to guarantee safe medication management for older people living at home. How robots for medication management influence the use of working time of home care professionals is one area that needs more investigation.

Our study aimed to examine the effect of using a robot for medication management on home care professionals’ use of working time. The research hypothesis was that using a robot for medication management would decrease the professionals’ use of working time in home care.

## Methods

### Study design

**Fig. 1:**
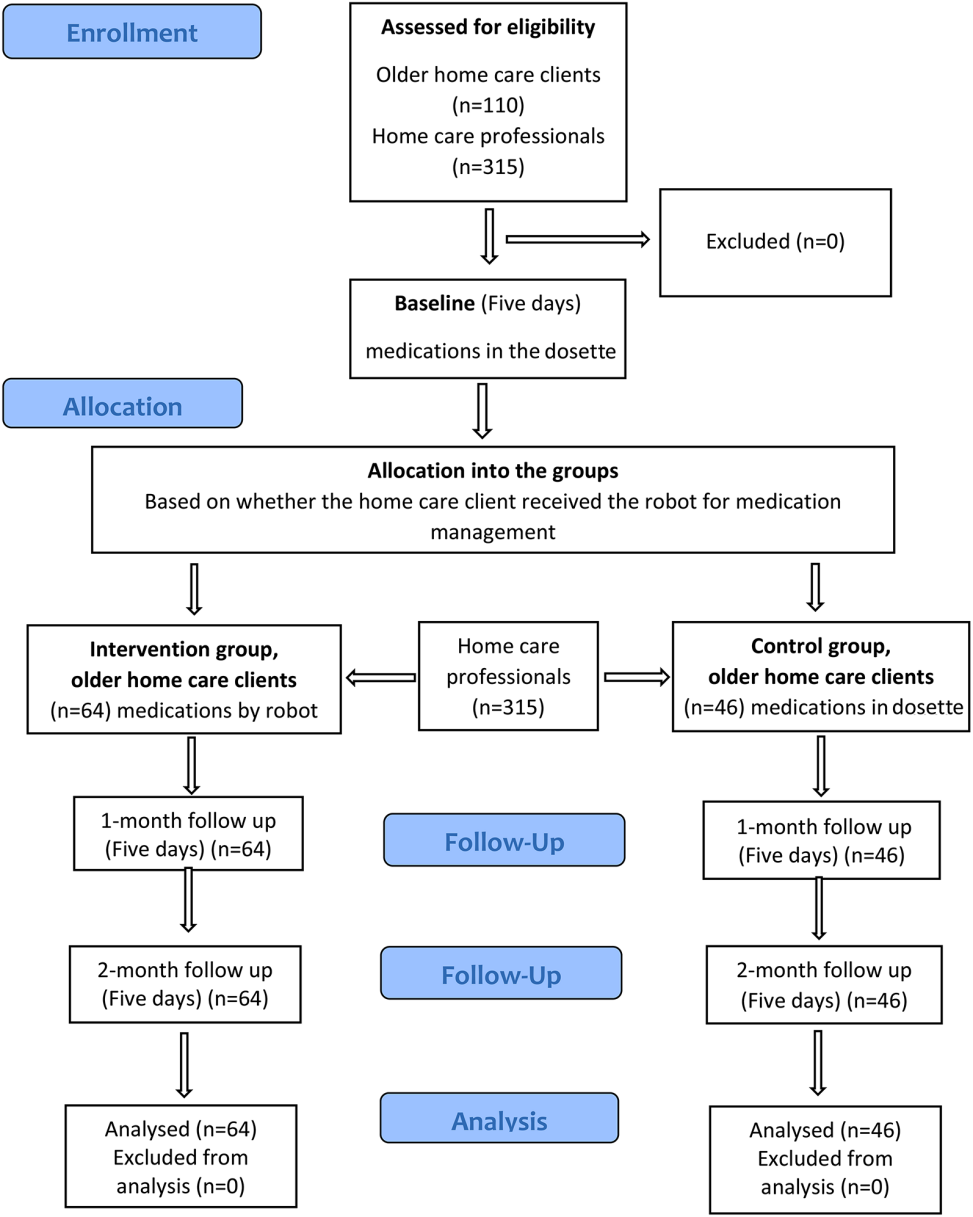
The CONSORT diagram of the study

A pragmatic non-randomized controlled clinical trial with three data collection points (at baseline, 1 month and 2 months) design [22] was carried out in Finland in 2021 (Fig. 1). It was designed to evaluate the effectiveness of an intervention under real-world conditions, provide a more accurate picture of how treatments work in practice, and help improve the quality and effectiveness of healthcare delivery [23]. The study was registered retrospectively (18/06/2023) on Trial registration (ClinicalTrials.gov Identifier: NCT05908604). The CONSORT Checklist was used for reporting the study [24].

### Research environment

This study was conducted in home care in Eastern Finland. Home care there is organized with different service providers, such as municipal home care services [25, 26], private and third sector services [27, 28]. Municipal home care services consist of home visits, including support for everyday activities and self-care, and counselling on the services available [25, 26]. The private sector services consist of assistance and home care services realized in clients’ homes with 24-hour assistance. The care and services provided by the third sector consist of day-to-day home care services in older clients’ homes [27, 28]. In this article, we use the term home care professional, which includes public health nurses, practical nurses, and registered nurses who are working in home care. In Finland, public health nurses and registered nurses complete their degrees at a University of Applied Sciences. The degree complies with the European Qualifications Framework (EQF) [29] and is defined as level six education with 210 ECTS and 240 ECTS. Practical nurses have completed level-four training (EQF) consisting of 180 ECTS on vocational qualification [30].

In this study, the collaboration organization operating in a rural region has 800 home care professionals with 1,500 older home care clients. In Finland, home care focused on care provided within older clients’ homes and consisted of several home care professionals with several clients. In practice, there is no own-nurse system and therefore, the different professionals visit the same clients’ homes. In the year 2021, the organization purchased 110 robots for medication management. The use of robots for medication management in home care has been implemented in some areas within the region, with the future goal of providing access to all that are able to use them. The aim is to decrease home care professionals’ home visits and increase older home care clients’ safe medication management [31]. Clients using the robots pay a monthly service fee based on their income as part of their home care services. For example, low-income clients have been able to get home care services for free including the robot. Medications are dispensed ready to home care clients in the pharmacy by the pharmacist and delivered in single-dose bags for two weeks. One single-dose bag contains medications for one intake. In the older home care clients’ homes, the professionals dispense medication manually to the dosette once a week and then visit up to even five times per day in clients’ homes to administer the medications. In Finland, the goal of home care for older clients is to support them to live independently at home as long as possible and therefore, several home visits per day are appropriate and common [21]. When using a robot, the professionals put the bags in the robot for two weeks. The robot stands on a table, assists older home care clients with spoken instructions and sound signals by dispensing their medications at the right time. In addition, the robot displays written instructions on the device screen using indicator lights. In addition, if the client does not take the medication after reminders, the robot is locked and only home care professionals can open it. Moreover, the robot allows home care professionals to monitor older home care clients’ medication management. (Please see the picture and more information: https://www.evondos.com/) [32].

### Study participants

The study participants were home care professionals including public health nurses, registered nurses, and practical nurses. Inclusion criteria for participation in this study were as follows: (1) voluntary participation in the study, (2) currently working in older people’s home care and (3) able to communicate in Finnish or English. One of the researchers (RT) received research permission from the participating organization. After that, the head of home care was contacted by one of the researchers (RT) to arrange meetings with the home care professionals. During the meetings, the researcher (RT) provided information about the study, including the information of inclusion criteria for study participants.

### Study conduct

The study was conducted in 2021. Home care professionals assessed clients’ ability to use the robot for medication management. They proposed the robot for the clients who: (1) had regular tablet-form medication in use, (2) were able to use the robot independently, and (3) chose to use the robot voluntarily. Home care professionals didn’t propose the robot for the clients who: (1) had physical challenges, such as poor eyesight, and/or (2) had neurological challenges, such as memory disorders. The home care team leader made the final decision about receiving the robots. Robots were given to those clients in the home care region that implemented the use of the robots who the professionals thought would be able to use them as the intervention group (IG). The control group (CG) consisted of clients living in the home care region that did not implement the use of the robots for medication management but who the professionals thought would have been able to use it.

The IG’s baseline periods were individually defined. For example, before the client in the IG group received a robot, a five-day baseline period was carried out. The next day after the five-day baseline period, the client started to use the robot for medication management. A common baseline period for all CG group clients was defined. The home care professionals who participated in the study made home visits to homes of both IG and CG group clients. The data were collected from April to November in 2021.

The non-random allocation into the IG and the CG was made before the baseline period (five days). In this pragmatic trial, random allocation was not feasible due to ethical and logistical considerations and limitations in resources for conducting the trial. Blinding was also impossible given the nature of the intervention. Altogether 64 home care clients were allocated to the IG and 46 to the CG (Fig. 1). In both groups, pharmacies dispensed medications that were to be taken regularly packed in single-dose bags to be sufficient for two weeks. After that, the pharmacies delivered the bags to home care services. In the IG, home care professionals loaded them inside the robot; this enabled older home care clients to carry out medication management by themselves. In the CG, home care professionals dispensed medications manually to the dosette box during home visits.

### Measures

The primary outcome measures were the total amount of home visits (frequency) during the entire intervention period and total working time of home care professionals used for medication management (in minutes) during the entire intervention period. The secondary outcome was the home care professionals’ working time used for medication management considering the number of visits per day. The data were collected using the Working Time Tracking Form developed by a professional team for this study [33]. The team included a senior lecturer in nursing with medication management competence, a head of older people’s home care, and a home care professional working in older people’s home care. The content of the Time Tracking Form was based on previous literature about medication management in home care [6, 7]. The research protocol was planned by the authors before the study; it can be accessed from the corresponding authors on a reasonable request. In the Working Time Tracking Form, the home care professionals recorded the time in minutes they used for each phase of the process, i.e., ordering medications, dispensing medications to the dosette or dispensing medications to the robot, reconstituting medications, administering medications as tablets, administering medications via other routes, medication education, and monitoring the effects of medications. The Working Time Tracking Form was filled for all clients in both IG and CG at every home visit involving medication management during a 5 day period (Monday to Friday) at baseline and at follow-ups at one and two months.

The Time Tracking Form was evaluated by the expert panel and pre-tested by a pilot group in terms of content validity [33]. The expert panel consisted of seven participants: one head of home care, three home care professionals, and two senior lecturers in nursing with medication management competence, and one statistician. The panel members evaluated each item focusing on usability, but they did not find any need for revision. This helped evaluate how understandable and clear the Time Tracking Form was and how long it took to complete it [34]. The answers of the pre-test were not included in this study.

### Data collection

Data were collected from the groups using the Working Time Tracking Form at three data collection points (baseline, months 1 and 2). In addition, home care clients’ gender and age were asked as background data. The researcher (RT) delivered the paper Time Tracking Forms to the home care contact person who gave to the home care professionals who, in turn, took the forms to clients’ homes and filled them during the time spent on medication management during each home visit. The data collection lasted five days, from Monday to Friday, when most medication management was realized. The home care professionals returned the forms to the home care contact person who then delivered them to the researcher.

### Data analysis

The SPSS v.26 software was used for data analysis by an expert statistician. The characteristics of the sample were reported using descriptive statistics including frequencies, percentages, mean values, and standard deviation.

A summation variable based on the items of the medication management process was developed to form total time for medication management. The homogeneity of groups referring to clients was tested using two-sample t-test (age) and chi-squared test (gender). The t-test was used to compare the groups at each of the three timepoints for each phase of the process. P-values were corrected with Bonferroni correction to avoid Type I error in inference (Table 2). Analysis of Covariance was used to examine differences (Sidak multiple comparisons) between timepoints within both groups for the total time for medications, with the number of visits per day as the covariate (Table 3). The p-value ≤ 0.05 was regarded as statistically significant. There were no missing data.

### Ethical considerations

The Ethics Committee of the University of Eastern Finland provided ethical approval (24/2017) and the participating healthcare organization granted the required research permission. The study followed ethical principles and all methods were performed in accordance with Ethical principles and the Declaration of Helsinki [35].

One of the researchers (RT) informed 352 home care professionals about the study. The information included the aim of the study and the data collection process. Furthermore, the home care-professionals were informed of voluntary participation and their right to discontinue their participation in the study at any time. Furthermore, the home-care professionals were informed about their anonymity to ensure privacy. Altogether 315 home care professionals agreed to participate in the study and during the study period, they were taking care of 110 home care clients. All participants signed the informed consent form when agreeing to participate. No compensation was paid to the participants and their employer for participation in the study.

## Results

### Demographic characteristics

The clients were mostly female in both groups (IG: n = 46, 71.9%; CG: n = 35, 76.1%, p = 0.621). Their mean age was 79.3 years, SD 6.4 (IG: 79.1 y, SD 6.6; CG: 79.6 y, SD 6.0, p = 0.718). There were no statistically significant differences between the groups. There was no attrition of samples and all recruited participants remained in the study until the end. In addition, no harm was reported of the intervention. The home care professionals’ demographic characteristics are presented in Table 1.

**Table 1:** Home care professionals’ background factors (n = 315)

Background factor	n (%)
**Occupation**	
Practical nurse	287 (91.1)
Registered nurse	22 (7.0)
Public health nurse	6 (1.9)
**Age**	
18–24 years	19 (6.0)
25–34 years	149 (47.3)
35–44 years	56 (17.8)
45–54 years	47 (14.9)
55 > years	44 (14.0)
**Gender**	
Male	13 (4.1)
Female	302 (95.9)
**Work experience in nursing**	
< 1 year	19 (6.0)
2–5 years	85 (27.0)
6–10 years	89 (28.3)
10 > years	122 (38.7)
**Work experience in older people’s home care**	
< 1 year	35 (11.1)
2–5 years	122 (38.7)
6–10 years	104 (33.0)
10 > years	54 (17.1)

### Total amount of home visits and working time used for medication management

In the IG group, the total amount of the home visits for 64 clients in a 5-day period decreased by 89.4% from baseline at the 1-month follow up, from 878 visits to 93 visits, and by 92.5% at the 2-month follow up, from 878 visits to 66 visits (p < 0.001). In the CG group, the total amount of home visits for 46 clients in a 5-day period remained almost the same from baseline (670 visits) to the 1-month follow-up (668 visits) and the 2-month follow-up (668 visits).

Home care professionals’ total working time for medication management increased from baseline to the 1-month follow-up and the 2-months follow-up (mean 6.49, 16.37, 16.74, respectively) in the IG group. In the CG group, the total working time for medication management remained nearly the same from baseline to the 1-month follow-up and the 2-month follow-up (mean 5.15, 4.48, 5.13, respectively). After robot use, home care professionals didn’t use working time for dispensing medications to the dosette, reconstitution medications, administrating medications as tablets or administering medications using other routes. After implementing the robot, home care professionals’ tasks related to medications focused on ordering medications, dispensing medications into the robot, monitoring the effects of medications, and medication education in the IG group. (Table 2).

**Table 2:** Working time used for medication management in minutes analyzed with two sample t-test (Bonferroni corrected p-values)

Variable	Baseline (5 days)			1-month (5 days)			2-month (5 days)		
	IG (n = 64) mean^a^ (SD)	CG (n = 46) mean^a^ (SD)	p-value	IG (n = 64) mean^a^ (SD)	CG (n = 46) mean^a^ (SD)	p-value	IG (n = 64) mean^a^ (SD)	CG (n = 46) mean^a^ (SD)	p-value
Total time for medication management	6.49 (2.7)	5.15 (3.1)	< 0.003	16.37 (5.73)	4.48 (2.92)	< 0.003	16.74 (4.36)	5.13 (3.32)	< 0.003
Ordering medications	0.03 (0.43)	0.4 (1.73)	< 0.003	0.05 (0.52)	0.23 (1.20)	0.039	0	0.60 (2.09)	< 0.003
Dispensing medications into the robot	0	0		8.43 (5.74)	0	< 0.003	10.77 (2.95)	0	< 0.003
Dispensing medications to the dosette	1.60 (1.46)	0.68 (1.61)	< 0.003	0	0.50 (1.68)	< 0.003	0	0.49 (1.68)	< 0.003
Reconstitution medications	0.63 (0.84)	0.32 (0.47)	< 0.003	0	0.09 (0.29)	< 0.003	0	0.09 (0.28)	< 0.003
Administrating medications (Tablet)	2.59 (1.14)	2.09 (1.18)	< 0.003	0	2.46 (1.37)	< 0.003	0	2.38 (1.46)	< 0.003
Administrating medications using other routes	0.43 (0.81)	0.83 (0.99)	< 0.003	0	0.64 (1.10)	< 0.003	0	0.66 (1.14)	< 0.003
Monitoring medications’ effects	0.39 (0.81)	0	< 0.003	0	0.0 (0.077)	1.00	0.18 (0.58)	0	< 0.003
Medication education	0.82 (1.21)	0.80 (1.54)	1.00	7.88 (3.09)	0.56 (1.23)	< 0.003	5.79 (2.54)	0.91 (1.58)	< 0.003

^a^Five-day mean in minutes per client

IG: Intervention Group; CG: Control Group; SD: Standard Deviation

The total working time used for medication management considering the number of visits per day decreased from 54.2 min (95% CI 49.6–58.8) to 34.9 min (31.4–38.3), i.e., by slightly over 19 min (p < 0.001) in the IG group. During the follow-up, the total working time used for medication management considering the number of visits per day remained the same in the CG group. (Table 3.)

**Table 3:** The total working time (in minutes) used for medication management considering the number of visits per day analyzed with analysis of covariance (Sidak multiple comparisons)

Variable	IG (n = 64)					CG (n = 46)				
	Baseline (T1) mean^a^ (95% CI)	1-month (T2) mean^a^ (95% CI)	2-months (T3) mean ^a^ (95% CI)	Difference T2-T1 mean^a^ (p-value)	Difference T3-T1 mean^a^ (p-value)	Baseline (T1) mean^a^ (95% CI)	1-month (T2) mean^a^ (95% CI)	2-months (T3) mean^a^ (95% CI)	Difference T2-T1 mean^a^ (p-value)	Difference T3-T1 mean^a^ (p-value)
The total working time used for medication management	54.2 (49.6–58.8)	40.8 (37.4–44.3)	34.9 (31.4–38.3)	-13.4 (< 0.001)*	-19.3 (< 0.001)*	75.2 (70.1–80.4)	65.0 (59.8–70.1)	74.3 (69.2–79.4)	-10.2 (0.02)*	-0.95 (0.992)

^a^Five-day Estimated Marginal Mean in minutes per group

Abbreviations: IG, Intervention Group; CG, Control Group; CI, Confidence Interval

## Discussion

This study aimed to examine the effect of using a robot for medication management on home care professionals’ use of working time. The study produced new knowledge about the effect of a digital solution on home care professionals’ use of working time for medication management. The total number of home visits decreased considerably in the IG group. This is because the home care professionals did not visit IG clients’ homes to administer medications, which is logical because the robot administrated the medications. However, the total time for medication management increased in the IG group. This is due to working time used for dispensing medications into the robot and medication education. It is noteworthy that after robot use, medication training included education about robot use for medication management. The clients might have needed education for robot use and not for medication management. More specifically, the home care professionals’ working time used for medication management concerning the number of visits per day decreased considerably. Thus, our results support our hypothesis that using a robot for medication management decreases the professionals’ use of working time in home care.

Without robot use for medication management, the medication process could be a time-consuming task in daily home care, as reported in previous studies [8, 9]. Furthermore, older home care clients have several medications [4, 5] and therefore, medications should be given at the right time [9, 10]. The growing number of older people increases the need for daily home care services and increases the cost of healthcare. In addition, this phenomenon causes a shortage of labor, including home care professionals [35–37]. Therefore, using a robot decreases professionals’ time for medication management and the time saved can be assigned to care and services that cannot be replaced otherwise. Our study showed that the use of a robot for medication management decreased considerably the number of home care professionals’ home visits. This means that the time savings have obvious benefits that should be quantified for the organization, such as cost effectiveness gains, for example average salary and work schedule.

Decreasing home visits due to the use of a robot for medication management raises ethical questions. For instance, robot use in home care can increase older home care clients’ loneliness [39]. Loneliness among older home care clients has been shown to have health-related, psychological, and social consequences including social isolation, mental illness and even nutritional risks [38, 39]. However, previous studies [3, 40, 41] have reported that robots for medication management improve older home care clients’ everyday life by increasing their autonomy. Moreover, clients emphasized that using the robot increased their safe medication management [42].

Based on previous studies, digital solutions for nursing care have influenced nurses’ working time [16, 17]. In our study, the robot for medication management influenced the working time of the home care professionals. The robot decreased especially the time used by home care professionals when administering medications to older home care clients. Most medication errors typically occur during the medication administration stage. Wrong medication, wrong dose, and wrong timing are the usual types of errors in home care that endanger patient safety [6, 7]. Therefore, using a robot for medication management supports older people’s independent living in their homes and enables the safety of medication management in collaboration with older home care clients, their relatives and home care professionals.

The results indicated that with the use of the robot for medication management, the time used for medication education increased. It occurred because after robot use, medication training included education about robot use for medication management. However, one essential question concerns the lack of medication education. Based on our results, in the CG group medication education decreased from baseline to 1-month and then increased from 1-month to 2-month effecting the total working time used for medication management. It might be that during the 1-month with a 5-day period, the clients didn’t have need for medication education or no new medications were prescribed for clients in the CG group and therefore, the time spent on medication education was lower.

Among older home care clients, it is evident that they have various chronic diseases [43] with multiple medication regimens. Therefore, home care professionals should pay more attention to medication education including the indications for medication, the schedule for taking medications, as well as common and severe adverse effects [13]. Medication-related errors, especially in clients with high-risk medications [14], cause serious consequences for older home care clients’ health and can lead to readmission to long-term healthcare settings, hospitalization and even death [3].

### Limitations and strengths of the study

Due to pragmatic reasons, we were unable to perform random allocation in our study. While random allocation is considered the gold standard for clinical trial design, we had to balance the need for rigorous scientific methodology with the practical realities of conducting a study in a real-world clinical setting. As a result, we had to use a non-randomized allocation method to assign participants to different groups, which causes limitations in terms of internal validity and generalizability of the findings. However, we believe that the pragmatic approach we used allowed us to better reflect the clinical practice and optimize the study’s feasibility and acceptability, thus providing insights that inform clinical practice and policy decisions. The Working Time Tracking Form was designed and used for the first time in this study. The home care professionals described the form as easy to use, but medication education in the IG at the follow-up assessment at 1 and 2 months might also include robot use guidance. For further use, it is suggested to revise this form to separate the medication education and robot use guidance. In addition, it is noteworthy that the filling of the Working Time Tracking Form affected the workload of the home care professionals, but based on their evaluation, it was on average 2 min per visit.

Moreover, the data were collected with a paper form in older home care clients’ homes. This avoided the risk of high attrition rates among home care professionals posed by electronic data collection after the home visits [44]. In addition, a systematic and researcher-informed data collection in collaboration with a contact person in home care was used to minimize the drop-out rate of home care professionals and thus, home care clients. Our results represent the implementation of the robot for medication management in the home care in one city. Thus, only preliminary conclusions and cautious generalizations can be made. However, to our knowledge, ours is the first study evaluating the effect of robot use on home care professionals’ use of working time from the perspective of the medication management process.

## Conclusions

Using a robot for medication management had a decreasing effect on home care professionals’ use of working time. Consequently, it can lead to better health outcomes, improved satisfaction among older clients, and a reduction in readmissions to healthcare settings. Additionally, it can also reduce home care professionals’ workload and stress levels and enhance their work efficiency, allowing them to complete their tasks with fewer errors, which leads to improved patient safety.

The knowledge produced in this study has implications for practice and research. Robot for medication management should be widely implemented based on older home care clients’ and professionals’ needs to meet the challenge of a growing number of older people in need of home care. Future research is needed to evaluate the cost-effectiveness when using a robot for medication management in older people’s home care. In addition, research focusing on medication incidents and medication adherence is needed to improve robot-based medication management.

## Data Availability

The datasets used and analyzed during the current study are available from the corresponding author on reasonable request.

## List of abbreviations

CG: Control group
CI: Confidence Interval
CONSORT: Consolidated Standards of Reporting Trials
ECTS: The European Credit Transfer and Accumulation System
EQF: the European Qualifications Framework
F: Frequency
IG: Intervention group
SD: Standard deviation
SPSS: Statistical Package for the Social Sciences

## References

[1] Zanjal S, Talmale G (2016). Medicine reminder and monitoring system for Secure Health using IOT. Procedia Comput Sci.

[2] Puustinen J, Kangasniemi M, Turjamaa R (2020). Are comprehensive and individually designed care and service plans for older people’s home care a vision or a reality in Finland?. Health Soc Care Community.

[3] Mira J (2019). Medication errors in the older people population. Expert Rev Clin Pharmacology.

[4] Rochon PA. Drug prescribing for older adults. https://www.uptodate.com/contents/drug-prescribing-for-older-adults.

[5] Lagerin A, Törnkvist L, Nilsson G, Johnell K, Fastbom J (2020). Extent and quality of drug use in community-dwelling people aged ≥ 75 years: a Swedish nationwide register-based study. Scand J Public Health.

[6] Karttunen M, Sneck S, Jokelainen J, Männikkö N, Elo S (2019). Safety checks, monitoring and documentation in medication process in long-term elderly care–nurses’ subjective perceptions. J Nurs Educ Pract.

[7] Lorenzini C (2021). Managing multiple medications and their packaging for older people in Home Care nursing: an interview study. Healthcare.

[8] Berland A, Bentsen SB (2017). Medication errors in home care: a qualitative focus group study. J Clin Nurs.

[9] Lindblad M, Flink M, Ekstedt M (2017). Safe medication management in specialized home healthcare - an observational study. BMC Health Serv Res.

[10] National Institute for Health and Welfare (2015). Turvallinen lääkehoito: Opas lääkehoitosuunnitelman tekemiseen terveydenhuollossa.

[11] Vaismoradi M, Jamshed S, Lorenzl S, Paal P (2021). PRN Medicines Management for Older People with Long-Term Mental Health Disorders in Home Care. Risk Manag Healthc Policy.

[12] Strampelli A, Cerreta F, Vučić K (2020). Medication use among older people in Europe: implications for regulatory assessment and co-prescription of new medicines. Br J Clin Pharmacol.

[13] Bowen JF, Rotz ME, Patterson BJ, Sen S (2017). Nurses’ attitudes and behaviors on patient medication education. Pharm Pract.

[14] Lang A, Macdonald M, Marck P, Toon L, Griffin M, Easty T, Fraser K, MacKinnon N, Mitchell J, Lang E, Goodwin S (2015). Seniors managing multiple medications: using mixed methods to view the home care safety lens. BMC Health Serv Res.

[15] Kleiven H, Ljunggren B, Solbjør M (2020). Health professionals’ experiences with the implementation of a digital medication dispenser in home care services – a qualitative study. BMC Health Serv Res.

[16] Turjamaa R, Kapanen S, Kangasniemi M (2020). How smart medication systems are used to support older people’s drug regimens: a systematic literature review. Geriat Nurs.

[17] Kangasniemi M, Karki S, Colley N, Voutilainen A. The use of robots and other automated devices in nurses’ work: An integrative review. Int J Nurs Pract. 2019;25(4), e12739. 10.1111/ijn.12739 31069892

[18] Rantanen P, Parkkari T, Leikola S, Airaksinen M, Lyles A. An In-home Advanced Robotic System to Manage Elderly Home-care Patients’ Medications: A Pilot Safety and Usability Study. Clin Ther 2017;39(5), 1054–1061. Available form: 10.1016/j.clinthera.2017.03.020. 10.1016/j.clinthera.2017.03.020 28433400

[19] Martini N, Broadbent E, Koo J, Lam L, Verches D, Zeng S, Montgomery-Walsh R, Sutherland C. Investigating the Usability, Efficacy and Accuracy of a Medication Entering Software System for a Healthcare Robot. Front Robot AI 2022;(25)9,814268. Available from: 10.3389/frobt.2022.814268. 10.3389/frobt.2022.814268 PMC8821944 35146001

[20] Eurostat (Producer). Ageing Europe – Looking at the lives of older people in the EU 2019. Available from: https://ec.europa.eu/eurostat/statisticsexplained/index.php?title =Ageing_Europe_-_looking_at_the_lives_of_older_people_in_the_EU.

[21] Ministry of Social Affairs and Health. Quality recommendation to guarantee a good quality of life and improved services for older persons 2020–2023. 2020. Available from: http://urn.fi/URN:ISBN978-952-00-8427-1.

[22] Cuello-Garcia C, Santesso N, Morgan R, Verbeek J, Thayer K, Ansari M, Meerpohl J, Schwingshackl L, Katikireddi S, Brozek J, Reeves B, Murad M, Falavigna M, Mustafa R, Regidor D, Alexander P, Garner P, Akl E, Guyatt G, Schünemann H (2022). GRADE guidance 24 optimizing the integration of randomized and non-randomized studies of interventions in evidence syntheses and health guidelines. J Clin Epidemiol.

[23] Gamerman V, Cai T, Elsäßer A (2019). Pragmatic randomized clinical trials: best practices and statistical guidance. Health Serv Outcomes Res Method.

[24] Altman DG (1996). Better reporting of randomised controlled trials: the CONSORT statement. BMJ.

[25] Health care act (1326/. 2010). https://www.finlex.fi/fi/laki/ajantasa/2010/20101326.

[26] Social welfare act (710/. 1982). https://www.finex.fi/fi/laki/alkup/1982/19820710.

[27] Private health care act 152/. 1990. http://www.finlex.fi/fi/laki/ajantasa/1990/19900152.

[28] Private social services act 922/. 2011. http://www.finlex.fi/fi/laki/alkup/2011/20110922.

[29] European Union. Description of the eight EQF levels. https://europa.eu/europass/en/description-eight-eqf-levels.

[30] Gobbi M, Kaunonen M, editors. Tuning Educational Structures in Europe: Guidelines and Reference Points for the Design and Delivery of Degree Programmes in Nursing. https://www.calohee.eu/wp-content/uploads/2018/11/WP-4-Del.-1.4-Guidelines-and-Reference-Points-for-the-Design-and-Delivery-of-Degree-Programmes-in-Nursing-FINAL-28NOV18.pdf.

[31] Ministry of Social Affairs and Health. Quality recommendation to guarantee a good quality of life and improved services for older persons 2020–2023. http://urn.fi/URN:ISBN978-952-00-8427-1.

[32] Evondos. https://www.evondos.com/.

[33] Rattray J, Jones MC (2007). Essential elements of questionnaire design and development. J Clin Nurs.

[34] Polit DF, Beck CT (2020). Nursing research: Generating and assessing evidence for nursing practice.

[35] World Medical Association. The World Medical Association Declaration of Helsinki. Ethical principles for medical research involving human subjects. https://www.wma.net/policies-post/wma-declaration-of-helsinki-ethical-principles-for-medical-research-involving-human-subjects/. 10.1191/0969733002ne486xx 16010903

[36] Dugstad J, Eide T, Nilsen ER, Eide H (2019). Towards successful digital transformation through co-creation: a longitudinal study of a four-year implementation of digital monitoring technology in residential care for persons with Dementia. BMC Health Serv Res.

[37] Dostálová V, Bártov A, Bláhová H, Holmerová I (2021). The needs of older people receiving home care: a scoping review. Aging Clin Experimental Res.

[38] Tomstad S, Dale B, Sundsli K, Sævareid HI, Söderhamn U (2017). Who often feels lonely? A cross-sectional study about loneliness and its related factors among older home-dwelling people. Int J Older People Nurs.

[39] Donavan N, Blazer D (2020). Social isolation and loneliness in older adults: review and commentary of a national academies report. Am J Geriatr Psychiatry.

[40] Jacobs G (2018). Patient autonomy in home care: nurses’ relational practices of responsibility. Nurs Ethics.

[41] Edgren J, Penttinen L, Mäkelä M, Asikainen J, Gerasin A, Havulinna S. Older clients’ resources towards to rehabilitation are often left un-utilized. Finnish Institute for Health and Welfare. Helsinki. http://urn.fi/URN:ISBN978-952-343-703-6.

[42] Turjamaa R, Vaismoradi M, Kangasniemi M (2022). Older home care clients’ experiences of digitalisation: a qualitative study of experiences of the use of robot for medicines management. Scand J Caring Sci.

[43] World Health Organization. Ageing: Healthy ageing and functional ability. https://www.who.int/westernpacific/news/q-a-detail/ageing-healthy-ageing-and-functional-ability.

[44] Cantrell MA, Lupinacci P (2007). Methodological issues in online data collection. J Adv Nurs.

