# Correction: Effect of robot for medication management on home care professionals’ use of working time in older people’s home care: a non-randomized controlled clinical trial

**DOI:** 10.1186/s12913-024-10584-1

**Published:** 2024-01-15

**Authors:** Satu Kajander-Unkuri, Mojtaba Vaismoradi, Jouko Katajisto, Mari Kangasniemi, Riitta Turjamaa

**Affiliations:** 1 Department of Nursing Science, Faculty of Medicine, University of Turku, Turku, Finland; 2 Diaconia University of Applied Sciences, Helsinki, Finland; 3 Faculty of Nursing and Health Sciences, Nord University, Bodø, Norway; 4 Department of Mathematics and Statistics, University of Turku, Turku, Finland; 5 Satasairaala, Pori, Finland; 6 Savonia University of Applied Sciences, Kuopio, Finland; 7 Faculty of Science and Health, Charles Sturt University, Orange, NSW Australia

**Correction to: BMC Health Serv Res 23, 1344 (2023)**

**https://doi.org/10.1186/s12913-023-10367-0**

Following publication of the original article [1], the authors identified errors in the Abstract, the Results section and Table 3.

Corrections (marked in **bold**):

1. Abstract (Results subsection): The total working time used for medication management considering the number of visits per day decreased from 54.2 min (95% CI 37.4–44.3 **49.6–58.8**) to 34.9 min (31.4–38.3), i.e., by slightly over 19 min (*p* < 0.001) in the IG group.
2. Results (‘Total amount of home visits and working time used for medication management’ subsection): The total working time used for medication management considering the number of visits per day decreased from 54.2 min (95% CI 37.4–44.3 **49.6-58.8**) to 34.9 min (31.4– 38.3), i.e., by slightly over 19 min (*p* < 0.001) in the IG group.
3. Table 3 (2nd column, 3.^rd^ row): 54.2 (37.4–44.3 **49.6-58.8**)

The original article [1] has been corrected.
